# Wheat physiology predictor: predicting physiological traits in wheat from hyperspectral reflectance measurements using deep learning

**DOI:** 10.1186/s13007-021-00806-6

**Published:** 2021-10-19

**Authors:** Robert T. Furbank, Viridiana Silva-Perez, John R. Evans, Anthony G. Condon, Gonzalo M. Estavillo, Wennan He, Saul Newman, Richard Poiré, Ashley Hall, Zhen He

**Affiliations:** 1 ARC Centre of Excellence for Translational Photosynthesis, Research School of Biology. Australian National University, Canberra, ACT 2601 Australia; 2 Agriculture Victoria, 110 Natimuk Road, Horsham, VIC 3400 Australia; 3 CSIRO Agriculture and Food, PO Box 1700, Canberra, ACT 2601 Australia; 4 Australian Plant Phenomics Facility, Australian National University, Canberra, ACT 2601 Australia; 5 Department of Computer Science and Computer Engineering, La Trobe University, Bundoora, VIC 3086 Australia

**Keywords:** Wheat, Photosynthesis, Machine learning, Deep learning, Hyperspectral reflectance

## Abstract

**Background:**

The need for rapid in-field measurement of key traits contributing to yield over many thousands of genotypes is a major roadblock in crop breeding. Recently, leaf hyperspectral reflectance data has been used to train machine learning models using partial least squares regression (PLSR) to rapidly predict genetic variation in photosynthetic and leaf traits across wheat populations, among other species. However, the application of published PLSR spectral models is limited by a fixed spectral wavelength range as input and the requirement of separate custom-built models for each trait and wavelength range. In addition, the use of reflectance spectra from the short-wave infrared region requires expensive multiple detector spectrometers. The ability to train a model that can accommodate input from different spectral ranges would potentially make such models extensible to more affordable sensors. Here we compare the accuracy of prediction of PLSR with various deep learning approaches and an ensemble model, each trained and tested using previously published data sets.

**Results:**

We demonstrate that the accuracy of PLSR to predict photosynthetic and related leaf traits in wheat can be improved with deep learning-based and ensemble models without overfitting. Additionally, these models can be flexibly applied across spectral ranges without significantly compromising accuracy.

**Conclusion:**

The method reported provides an improved prediction of wheat leaf and photosynthetic traits from leaf hyperspectral reflectance and do not require a full range, high cost leaf spectrometer. We provide a web service for deploying these algorithms to predict physiological traits in wheat from a variety of spectral data sets, with important implications for wheat yield prediction and crop breeding.

**Supplementary Information:**

The online version contains supplementary material available at 10.1186/s13007-021-00806-6.

## Introduction

The global population is estimated to reach 9.7 billion by 2050 [1]. As a result, the projected demand for cereal grain exceeds the agricultural forecast output [2]. World-wide crop production must double to satisfy projected global food demand [3]. Wheat is the second most important source of calories consumed globally after rice [4, 5]. The improvement of wheat yields in the face of reductions in arable land area and deleterious effects of climate change is paramount. Increasing biomass production and yield potential through increases in photosynthetic performance has become a major recent target for cereals breeding [6, 7]. However, physiological breeding for photosynthesis and related traits is hampered by the lack of high throughput screening tools to enable either selection of superior germplasm or genetic mapping of these traits in large populations [6–9].

Recently we developed a machine learning framework based on Partial Least Squares Regression (PLSR) and hyperspectral reflectance, which enables prediction of several physiological traits related to photosynthetic performance in wheat leaves with high accuracy and speed (30 s to 1 min per leaf; [4, 10]). Measuring photosynthesis-related traits, such as nitrogen per unit leaf area (N_area_) and leaf dry mass per area (LMA), require laborious, destructive, and expensive laboratory-based methods which may take several days. Similarly, the estimation of physiological traits underpinning photosynthetic capacity, such as maximum Rubisco activity normalised to 25 °C (*V*_*cmax25*_) and electron transport rate (*J*), require time-consuming gas exchange measurements (up to 20 min per derived value; see [11, 12]) and specific expertise.

Most prior work [6, 8, 10, 11, 13–21] on mapping hyperspectral reflectance based measurements of various plants to physiological traits and leaf biochemistry uses PLSR to develop predictive algorithms. PLSR has been used for studying diverse traits such as sucrose, reducing sugar and total sugar dynamics [18], leaf water status [17, 19], salinity stress [20] and leaf nutrient contents [21]. Diverse species studied include tobacco [6, 8], tree species [10, 13], soybean [11], maize [14], wheat [15], rice [20], okra [16] and mango [21]. Data sets used are usually small (i.e., just a few hundred samples) in most studies, hence resulting in overfitting to the training data in the model, which results in prediction within the training data but not in unseen samples, is a major problem that needs to be avoided. PLSR has been a popular method for spectral modelling because it is computationally simple and therefore effective at avoiding overfitting.

This paper explores various deep learning approaches for predicting leaf physiological traits in wheat and compares the results to the PLSR method. While deep learning has commonly been used for machine vision applications such as feature extraction and plant classification [16, 22, 23], it does not seem to have been exploited to explore natural variation in predicted leaf and physiological traits. However, photosynthetic traits have been extracted using artificial neural networks from data collected from transgenic tobacco canopies presenting a range of photosynthetic capacity generated by genetic engineering [8].

There are several advantages to using deep learning approaches for predicting leaf traits from leaf reflectance. First, existing PLSR-based methods require a fixed length hyperspectral reflectance spectral wavelength range as input, necessitating a separate model built for each trait and wavelength range. Second, published PLSR models for photosynthetic traits use reflectance spectra from the short-wave infrared range, requiring expensive multiple detector spectrometers [24]. Thus, the ability to train a model that can accommodate input from multiple spectroradiometers of different wavelength ranges would make such models extendable to a range of affordable sensors, including Vis–NIR imaging sensors. Third, it is possible that deep learning or an ensemble model could improve prediction accuracy over a PLSR modelling approach at the leaf level (as observed in [8]).

Here, we compare different machine deep learning algorithms to improve the prediction of physiological traits using wheat leaf reflectance spectral data published in Silva-Perez et al. [4, 12]. We conducted a thorough architecture search for the best deep learning algorithm comparing multi-layered perceptron (MLP), recurrent neural networks and 1D convolutional neural networks (CNN) and an ensemble model. Our multi-task deep learning approach predicts multiple traits using a single model. The model exploits correlations between traits to improve prediction accuracy.

To make our models more accessible to researchers and potentially wheat breeders, we have created a website (Wheat Physiology Predictor (shinyapps.io)) that hosts our pre-trained models. Users can upload their wheat hyperspectral reflectance measurements and our web server will return all physiological traits predicted by a selected model.

## Materials and methods

### Dataset description

We used the large multi-site, multi-environment wheat data set including two treatment regimes, collected by Silva-Perez et al. [4, 12] for the construction of the models. This dataset consisted of the entire hyperspectral reflectance spectra (400–2400 nm) from wheat leaves and the corresponding physiological traits concurrently measured on the same leaf section. Hyperspectral reflectance along with physiological, biochemical and morphological leaf traits were measured at various developmental stages in 67 wheat genotypes and nine triticale genotypes grown in the field in both Australia (35°16′18.8′′S 149°06′50.3′′E) and Mexico (27°22′15.0′′N 109°55′49.3′′W) and in the glasshouse in Australia under two nitrogen treatments. Experiments used in the current work are Aus1, Aus2, Aus3, and Mex1 described in Silva-Perez et al. [12].

This extensive data set included both measured data and parameters derived from biochemical modelling for the spectral models described here. Single point traits included CO_2_ assimilation rate (*A*, µmol CO_2_ m^−2^ s^−1^) and stomatal conductance (g_s_, mol H_2_O m^−2^ s^−1^) obtained from leaf gas-exchange measurements with a LI-COR (LI-6400XT) under an irradiance of 1800 mmol quanta m^−2^ s^−1^. Modelled traits included maximum velocity of Rubisco carboxylation (*V*_*cmax*_, µmol CO_2_ m^−2^ s^−1^), maximum velocity of carboxylation at 25 °C (*V*_*cmax25*_, µmol CO_2_ m^−2^ s^−1^), electron transport rate (*J*, µmol e^−^ m^−2^ s^−1^), *V*_*cmax25*_/ N_area_ (µmol CO_2_ s^−1^(g N^−1^)) derived from modeling of CO_2_ response curves of *A* using the C_3_ biochemical model of photosynthesis [25]. *V*_*cmax*_ was the trait most sensitive to temperature and we have addressed this issue by normalisation to 25 °C [26]. Leaf structural traits included leaf dry mass per area (LMA, g m^−2^), leaf nitrogen concentration (N_mass_, mg N g^−1^), leaf nitrogen per unit area (N_area_, g N m^−2^) and SPAD as a surrogate for chlorophyll content. Complete measurement protocols can be found in Silva-Perez et al. [12]. The relationships between these traits, their heritability and genetic component of their variation is described elsewhere [12].

**Fig. 1:**
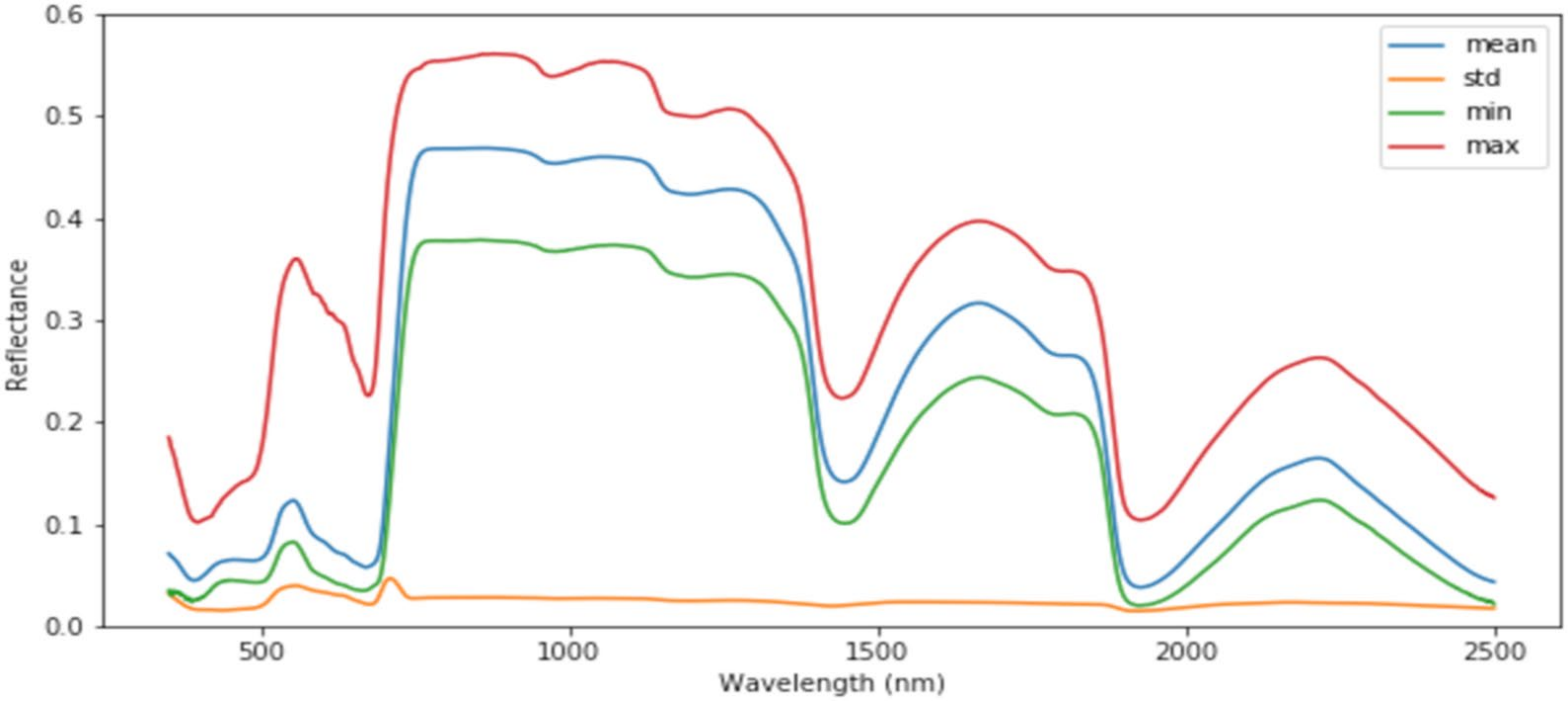
The mean, standard deviation, min and max reflectance measurements for the entire dataset used to build models graphically represented

Hyperspectral reflectance was measured with the Analytical Spectral Devices FieldSpec3 using a modified leaf clip containing an integrated light source described in Silva-Perez et al. [4]. Best practice for leaf spectral data collection and spectral modelling is described in [27] and lack of significant effects of measurement conditions inside the leaf clip-on data models is reported in [28]. In Aus1, Aus2, and Aus3 experiments, the reflectance was corrected with ‘jump correction’ at 1000 nm and 1800 nm, and in Mex1, reflectance was corrected at 1000 nm and 1830 nm using the software Spectral Analysis and Management System (SAMS^©^ The Regents of the University of California; https://github.com/carueda/sams). Reflectance values from all experiments were filtered from 400 to 2400 nm, and spectra with reflectance values at 800 nm lower than 0.35 and higher than 0.6 were deleted and treated as outliers (Fig. 1). The resulting data shows distinct regions of the spectrum with high reflectance. Our deep learning models were built to capture this distribution and map any systematic deviations from this distribution to corresponding physiological trait values. As in Silva-Perez et al. [4], we restricted the spectral range of the inputs to 400–2400 nm because the signal to noise ratio of the values outside this range was poor, possibly due to technical limitations of the radiometer.

Table 1 shows the statistical distribution for the total dataset of measured trait values used for model construction. The number of samples for each trait was different, between 488 and 1013, due to variable field conditions, which caused some measurements to be unusable. Details of these data are described in [4]. We randomly split the data for each trait into groups, with 70% for training, 10% for validation and 20% for testing. Samples were randomly assigned to these groups irrespective of experiment location or genotype. As this is a relatively small sample size for training deep learning algorithms, we used a number of strategies to overcome overfitting problems (see below).

**Table 1:** Summary of the statistical distribution of physiological traits used for model building

Trait	Samples No.	Mean	Median	STD
LMA	525	59.32	60.24	12.54
N_area_	1013	2.59	2.7	0.77
SPAD	614	48.5	49.75	8.27
N_mass_	615	42.89	43.84	8.6
*V*_*cmax*_	488	170.36	159.16	65.78
*V*_*cmax25*_	488	148.02	151.02	40.1
*J*	488	218.17	222.8	56.2
*A*	488	24.99	26.58	6.4
g_s_	488	0.48	0.45	0.21
*V*_*cmax25*_/N_area_	488	59.95	59.68	14.04

### Model building

The main challenge in mapping the hyperspectral reflectance data to physiological trait values is the long input sequence of 2000 individual wavelengths reflectance values. We considered the following three neural network architectures for modelling the input sequence: multilayer perceptron (MLP) [29]; recurrent neural networks in the form of long short term memory (LSTM) [30]; and 1D convolutional neural networks (CNNs) [31].

Each layer of an MLP essentially performs a linear transformation of the input to the output with a non-linear activation function applied after each layer. MLP’s contain at least three layers (input, hidden and output layers). There can be any number of hidden layers. This approach provides flexibility when mapping the input to the output; however, it cannot naturally find local spatial patterns that occur in multiple places in the input information.

Recurrent neural networks such as the LSTM are commonly used for modelling input text sequences for various natural language processing tasks. LSTMs can find temporal patterns in the input sequence. However, it is well known that LSTMs suffer from the “vanishing gradient problem”, which means they do not generally perform well for long input sequences [32]. Specifically, we trained a two-layered bi-directional LSTM model with 100 dimensional hidden units, which was then fed into a fully connected layer with 200-dimensional output and then finally into another fully connected layer that outputs the predicted trait value. We used a rectified linear activation function (ReLU) between the two fully connected layers [33]. Before feeding data into the LSTM model, we first perform global average pooling on every 10 input wavelengths, reducing the granularity of the data set.

A deep 1D convolutional neural network (1D CNN) can find a hierarchy of increasingly longer-range spatial patterns. At each layer, the 1D CNN slides a filter of learnt weights across the entire input length. This strategy of sliding the filter allows patterns found in one part of the sequence to detect patterns in other parts of it. The equation below shows the mathematical operation used to compute the 1D convolution for input X.

$$X_{{c}_{o} }^{(l)}= f\left({{\sum }_{{c}_{i}}{W}_{{c}_{o}}^{(l), {c}_{i}}}* {X}^{\left(l-1\right), {c}_{i}} + {B}_{{c}_{o}}^{l}\right).$$

where *l* denotes the *l*th layer of the CNN, *c*_*o*_ denotes the *c*_*o*_ th output channel, *c*_*i*_ represents the channel number of the input *X*^*(l−1)*^*,* *W*_*co*_^*(l),ci*^ is the convolutional kernel corresponding to the *c*_*i*_ th input channel and *c*_*o*_ th output channel, and *B*^*l*^_*co*_ is the learnable bias corresponding to the kernel of the *c*_*o*_th output channel, *f()* is the activation function (in our case ReLU was used) and * is element wise multiplication.

We used dilated 1D CNN layers to increase the receptive field size of the model. This allows each convolutional layer to see more of the input sequence, giving it a greater context to build its internal representation.

**Fig. 2:**
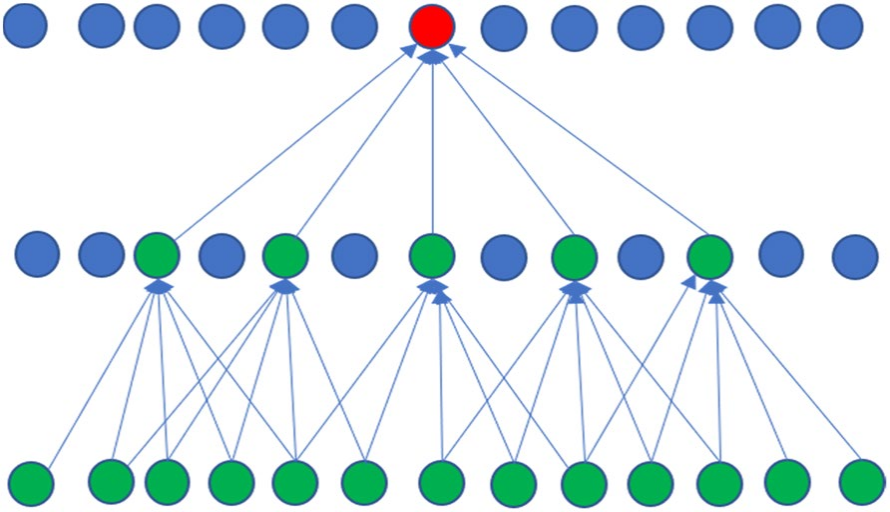
The dilated 1D CNN model parameters used here, namely a filter size of 5 and dilation factor of 1 for the first layer and dilation factor of 2 for the second layer. The figure illustrates the expanded receptive field (all green circles) of a single output neuron (red circle). So, each output neuron in this example depends on 13 input elements

**Fig. 3:**
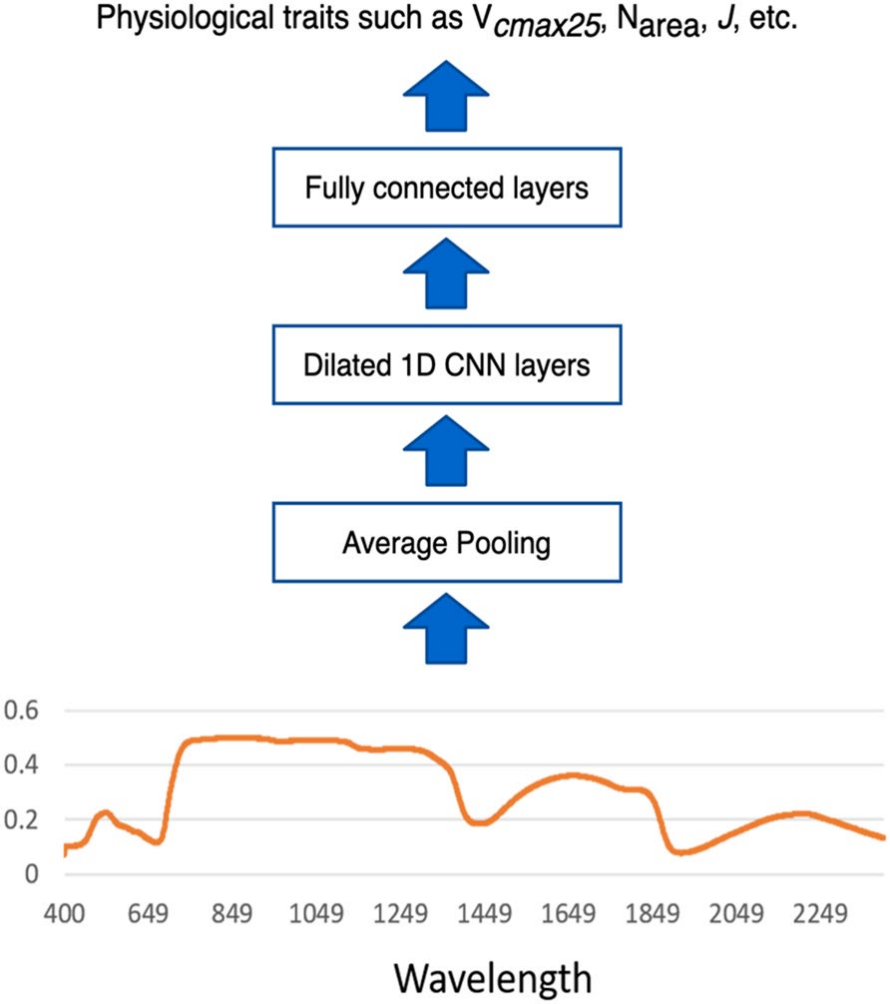
High level diagram of our neural network model architecture. Hyperspectral reflectance values are fed through sequential neural network layers, producing a scalar value for each predicted trait

A diagram illustrating the receptive field (green circles) of one output neuron (red circle) of a 2-layer 1D dilated CNN, which has a filter size of 5 and dilation factor of 1 for the first layer and dilation factor of 2 for the second layer is shown in Fig. 2. Figure 3 shows a higher level diagram of the neural network model architecture we have proposed for solving the problem of mapping hyperspectral reflectance to a physiological trait. First, average pooling is applied to the reflectance spectrum input to smooth the input signal since the detector can produce random fluctuations. Next, the dilated 1D CNN layers are used to extract the spatial patterns in the data. Between the 1D CNN layers we use batch normalisation and ReLU activations. Finally, an MLP consisting of fully connected layers are used to make the final prediction.

Table 2 shows the precise default model specifications used for the experiments.

**Table 2:** Architecture of the multi-task; single task models differ only in output size of the final fully connected layer (1 output unit instead of 10)

Type	Filter size	# Filters	Dilation	Batchnorm	Activation
1D average pool	10	–	–	–	
1D CNN	5	75	2	1D	ReLU
1D CNN	5	150	2	1D	ReLU
1D CNN	5	225	2	1D	ReLU
1D CNN	5	300	2	1D	ReLU
Flatten	–	–	–		
Fully connected	–	800	–	–	ReLU
Dropout (0.2 probability)	–	–	–	–	–
Fully connected	–	200	–	–	ReLU
Dropout (0.2 probability)	–	–	–	–	–
Fully connected	–	10 (multi-tasking) 1 (single tasking)	–	–	–

The loss function used for model optimisation was mean squared error (MSE), given by the following equation:

$$MSE \left(y, \widehat{y}\right)= \frac{1}{n}{\sum }_{i}^{n}{\left({y}_{i} - \widehat{{y}_{i}}\right)}^{2},$$

where y is the set of ground truth trait values and $\widehat{y}$ is the set of corresponding predicted values.

### Overfitting

Due to the relatively small data set size (around 350 training samples) per trait to be predicted, a major problem is overfitting to the training data and therefore producing a model that generalises very poorly to the test set or previously unseen data. We adopted three techniques to minimise this issue: early stopping, data augmentation and dropout [34].

Early stopping seeks to avoid overfitting to the training data set by stopping model training early in the training cycle. This method often reduces overfitting since the longer a model is trained, the more opportunity the model has to include noise in the input data to map to the output. Stopping early will usually result in the model preferentially mapping the relatively higher level characteristics of the input data to the output, which is likely to generalise better to the test data set [35]. In our case, we train a model for 1000 epochs, evaluating the validation data set every 10 epochs. The model with the highest validation score is retained throughout training; typically, this model is encountered much earlier than the end of training. The model that performs best on the validation set is most likely to be the best for generalising to the test set.

One way to artificially increase the size of the training data is to perform data augmentation. Training on additional augmented data can help a model better generalise the test data set by simulating random variations of the data. In particular, we perform random horizontal shifts (between − 5 and 5) on the hyperspectral reflectance data to boost the size of the data set by 50% from 772 to 1158 samples. A model trained on the augmented data set should generalise better to the test set because the data augmentation increases the variance in the training dataset and minimises the model's potential to overfit the training data.

We also used dropout before the final linear layer to help the model avoid overfitting the training data. Dropout randomly turns off a percentage of the neurons during training to prevent the neurons from co-adapting with each other from complex functions from the input to the output [34]. It, therefore, encourages the neurons to work more independently relative to each other and thus result in a simpler mapping from the input to the output.

### Multi-task learning

A single neural network can be trained to predict all the traits in one training run. This approach is called multi-tasking. The main benefit of this approach is that layers of the neural network can be trained, which are shared for predicting different traits. This effectively allows the model to exploit certain correlations between traits to refine the shared weights. Multi-tasking allows for model weights to be adjusted using multiple error signals from multiple loss values for a single training example. This is particularly useful given the small training data set since the combined losses from the different traits can help to avoid the model overfitting to noise in the values for any particular trait for a given example [36].

### Variable spectral ranges

**Fig. 4:**
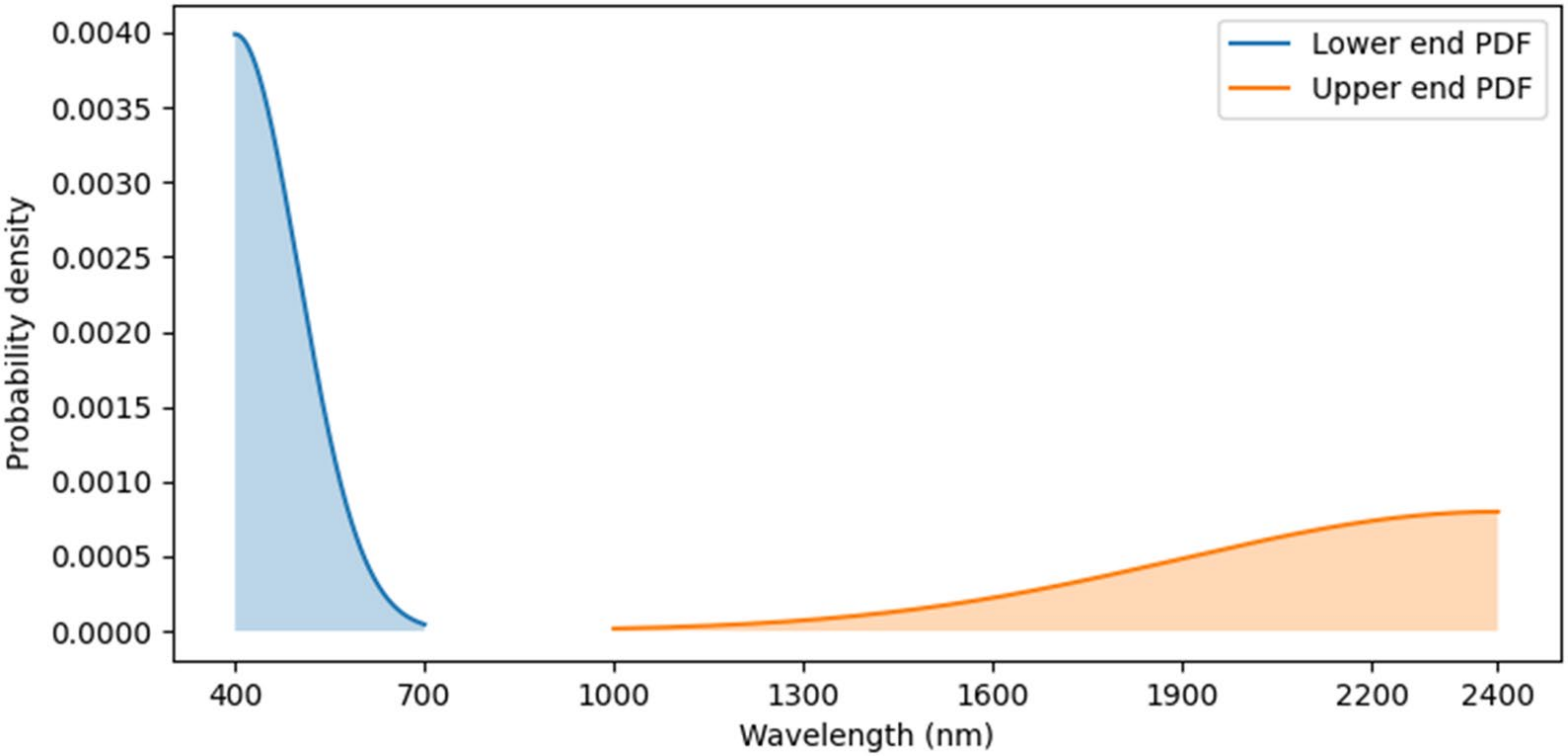
Probability density function (PDF) of the spectral trimming wavelength distributions

Hyperspectral reflectance measurement devices support a variety of spectral ranges, thus to maximise the usefulness of our trait prediction tool we sought to make models which support variable spectral ranges. Our primary training data consists of full-range spectroradiometer measurements [350 nm, 2500 nm], which we trim during pre-processing to 400–2400 nm to reduce noise components at the limits of detector range. As our CNN models require fixed-length input due to the linear layers at the output, we emulate variable spectral range inputs by way of data augmentation. We adopt a novel augmentation strategy dubbed “spectral trimming”, which trims both ends of the input spectrum randomly during training. Our strategy involves zeroing-out values at either end rather than trimming the input array to keep the inputs at a fixed length. More precisely, a pair of low/high wavelength values are randomly sampled for each training example, and reflectance values on the outside of this range are replaced with zeros. Both low and high values are sampled from separate truncated normal distributions with means of 400 nm and 2400 nm and standard-deviations of 100 nm and 500 nm, and are truncated such that their values are in [400 nm, 700 nm] and [1000 nm, 2400 nm], respectively (see Fig. 4). A further constraint is added to ensure that input examples retain a minimum range of 350 nm of valid (non-zero) values.

As this online augmentation technique is inappropriate for PLSR, we provide a further three PLSR models, each trained on a distinct spectral range dataset. The regular PLSR model is trained on the full [400 nm, 2400 nm] dataset, whereas the additional models are trained on the ranges [400 nm, 900 nm], [400 nm, 1000 nm], and [400 nm, 1700 nm] to align with spectral ranges of commonly available spectroradiometers. If reflectance data is uploaded that lies outside of these predetermined ranges, the input data is trimmed to match the nearest PLSR model.

### Model training details

Deep learning models were implemented using Pytorch and trained for 1000 epochs on a GTX 1080 TI graphics card. The specific variant of early stopping we used works as follows: the validation set is evaluated every 10 epochs, and the best performing model is retained. We used the Adam optimiser with an initial learning rate of 0.0001. The XGBoost and PLSR implementations used were from the XGBoost and SKLearn Python libraries, respectively. Both PLSR and XGBoost used fixed wavelenth ranges. The PLSR hyperparameters (number of components kept) was chosen by performing a search in the range of (1, 30) and choosing the value with the lowest mean validation R2 value. The XGBoost hyperparameters (learning rate, max depth, colsample_bytree) are chosen by performing a grid search over the values (0.01, 0.02, 0.05, 0.06, 0.08, 0.1), (3, 5, 7, 9, 11), (0.3, 0.5, 0.8, 1), respectively. These parameters were again chosen using the lowest mean validation R^2^. The full code of these models is located at https://github.com/ashwhall/hyperspec-trait-prediction.

### Wheat physiology predictor web server

As part of this work we have provided a publicly accessible web application (Wheat Physiology Predictor (shinyapps.io)) where users can upload wheat hyperspectral reflectance measurements in order to receive predicted physiological traits.

**Fig. 5:**
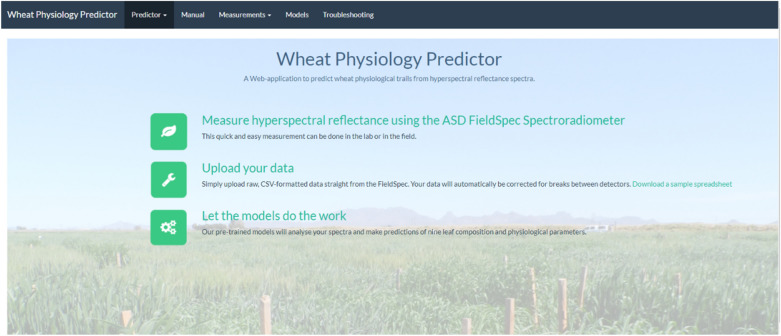
The home page of the Wheat Physiology Predictor

Figure 5 shows the home page of the website. The web server is written using the R Shiny R package that facilitates the building of interactive websites. The R Shiny server handles all data visualisation and user interaction. Behind the R Shiny web server is a Python server that implements the following models: Single task CNN, Multi-task CNN, PLS, and Ensemble. The single task CNN consists of 10 models each individually trained to predict a different trait. The multi-task CNN uses a single model to predict all trait values simultaneously. If PLS model is chosen, the input reflectance data is trimmed to the best-fitting range among the following options: [400, 900], [400, 1000], [400, 1700], [400, 2400], and the PLS model trained on the chosen range is used to predict the trait values. If the ensemble option is selected then the mean of all model predictions is returned.

The web site allows users to upload hyperspectral reflectance data for arbitrary wavelength ranges as input to the model, potentially accommodating a large range of spectroradiometers of different spectral ranges. To remedy the discontinuous jumps at the detector boundaries of multiple detector spectrometers, the website allows the user to specify the wavelengths at which these jumps occur and use ‘jump correction’ smoothing before model inference.

**Fig. 6:**
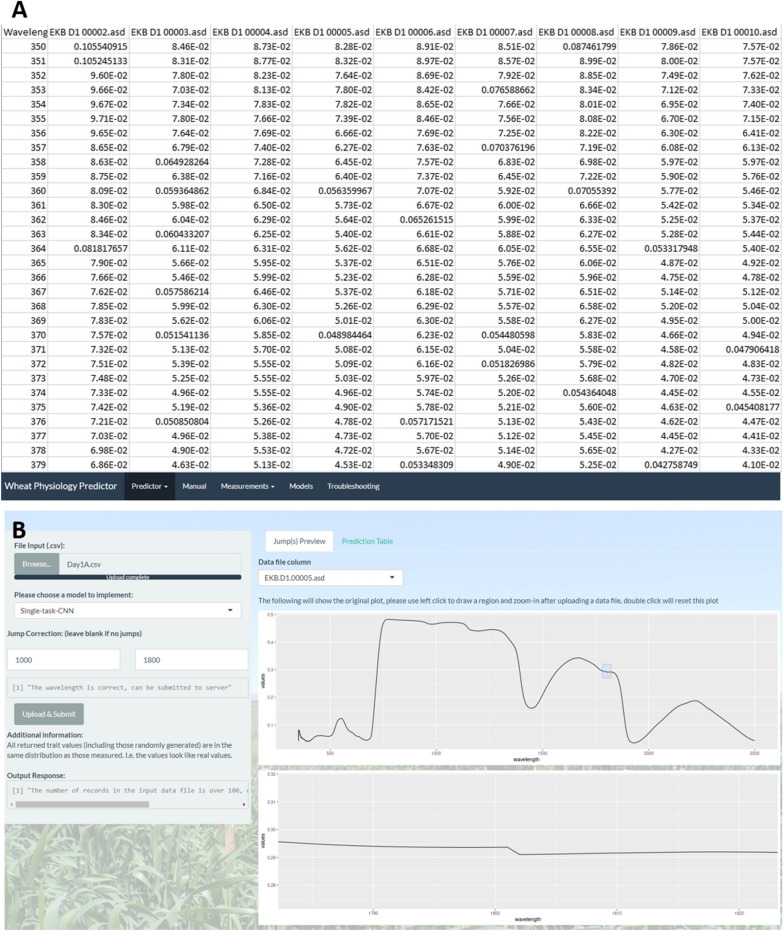
The input data file (**A**) and its preview plot (**B**) for the user to check the jump(s)

Figure 6 demonstrates an example for an input data file (6A) and the reflectance data is plotted at the first graph region (top 6B). After uploading a csv input file into the “Tool” tab of the R Shiny interface, it will automatically retrieve all the observations (columns in the csv file, excluding the first column which is the wavelength) and plot all the reflectance data for a specific observation at the “Jump(s) Preview” tab on the right, and the user can also select a specific observation from the dropdown list to preview the jumps occurred. The user can then draw a region onto the first plot and zoom-in that region at the second plot to accurately check the jumps at an arbitrary wavelength.

After checking the wavelengths at which the jumps occurred, the user can then input these wavelength values to the “Jump Correction” input fields on the left. This input field is constrained to numerical inputs and between 355 to 2495 nm. If an incorrect jump is specified, the text below will remind the user to correct it until it shows the correct information in Fig. 6.

**Fig. 7:**
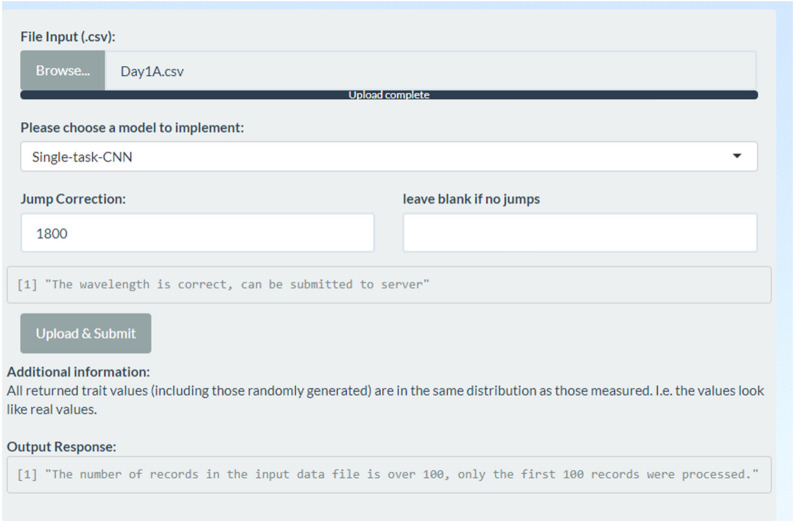
The output response warning on the number of observations from the server

The website allows users to upload a csv input file containing the measured hyperspectral wavelength values for up to 100 observations. The input file is required to have the following schema. The first column of the file must contain numerical wavelengths in increments of 1 nm, and the first row must be the name of each observation, thus each column contains the reflectance data for a single observation. Trait predictions are made for only the first 100 observations in the input file to maximise computational resource sharing among simultaneous users. The user is warned that only the first 100 observations are processed if they provide more than 100 observations, as shown in Fig. 7.

**Fig. 8:**
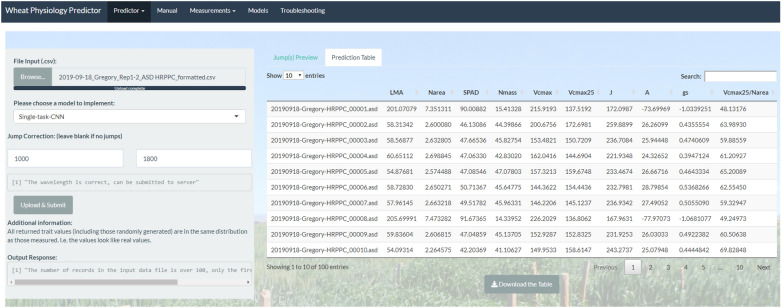
The results of the trait values delivered by the model prediction. The Python model server performs all model computations using CPUs. All models just take at most a few seconds to compute the required results due to the relatively small size of the models

The R Shiny server checks that the input files conform to the schema mentioned above. If the file conforms, the server forwards the input file to the Python model server to compute the predicted trait values. The predicted traits: LMA, N_area_, SPAD, N_mass_, *V*_*cmax*_, *V*_*cmax25*_, *J*, *A*, g_s_ and *V*_*cmax25*_/N_area_ (abbreviated as described above) are based on Silva-Perez et al. [4]. The user can export the results of the model predictions (Fig. 8) into a csv file by clicking the “Download the Table” button.

## Results

### Model performance

Using the trait data and reflectance information across the complete 400 to 2400 nm spectrum from Silva-Perez et al. [4, 12], we compared results from the multi-task 1DCNN, single-task 1DCNN, MLP, LSTM, XGBoost, PLSR and an ensemble model of both 1DCNNs and PLSR (Table 3).

**Table 3:** R^2^ for performance of various models on the test set

Model	LMA	N_area_	SPAD	N_mass_	*V*_*cmax*_	*V*_*cmax25*_	*J*	*A*	g_s_	*V*_*cmax25*_/N_area_	Mean
Multitask 1DCNN*	0.867	0.931	0.833	0.807	0.781	0.779	0.858	0.730	0.502	0.480	0.757
Multitask 1DCNN, No Spectral Trim	0.887	0.946	0.857	0.855	0.776	0.770	0.863	0.762	0.526	0.521	0.776
Singletask 1DCNN*	0.855	0.955	0.866	0.715	0.796	0.740	0.846	0.689	0.496	0.444	0.740
Singletask 1DCNN, No Spectral Trim	0.880	0.964	0.860	0.799	0.809	0.763	0.849	0.710	0.499	0.493	0.763
MLP	0.856	0.912	0.862	0.713	0.752	0.721	0.791	0.672	0.423	0.492	0.719
LSTM	0.704	0.809	0.748	0.685	0.578	0.640	0.732	0.615	0.352	-0.067	0.580
PLSR*	0.885	0.944	0.841	0.789	0.770	0.656	0.853	0.667	0.427	0.576	0.741
XGBoost	0.797	0.941	0.824	0.734	0.632	0.674	0.775	0.657	0.353	0.234	0.662
Ensemble	0.895	0.959	0.866	0.832	0.822	0.762	0.876	0.751	0.521	0.579	0.785

The results reported are the mean of three runs with different random seeds. Those marked with an asterisk are included in the ensemble model

While model performance varied depending on the predicted trait, the ensemble model performed better than PLSR alone for all traits. In contrast, the next best performing model across all traits was the multitask 1DCCN. As observed in Silva-Perez et al. [4], leaf mass per area (LMA) and leaf nitrogen per area (N_area_) could be predicted with the highest accuracy of all traits, whereas stomatal conductance (*g*_s_) and maximal Rubisco activity per unit leaf nitrogen (*V*_*cmax25*_/N_area_) were the most challenging regardless of the model used. As expected, due to our input data comprising 2000 wavelengths of light, LSTM did notpredict wel across the majoprity of traits.

Table 4 explores bias in predictions derived from the models tested and Table 5 REP for these same models on the test set. The absolute value of the bias between the predicted and actual trait values is reported to ensure that mean value accurately portrays the magnitude of the bias. The multitask 1DCNN exhibits the smallest bias, followed by the ensemble of models.

**Table 4:** Abs(Bias (%)) for various models on the test set

Model	LMA	N_area_	SPAD	N_mass_	*V*_*cmax*_	V_cmax25_	*J*	*A*	g_s_	*V*_*cmax25*_/N_area_	Mean
Multitask 1DCNN	0.754	0.737	1.150	0.556	1.502	0.614	0.866	0.817	2.683	0.905	1.058
Multitask 1DCNN, No Spectral Trim	0.704	1.105	0.574	1.001	2.619	2.359	1.878	2.390	3.842	0.678	1.715
Singletask 1DCNN	2.307	1.242	0.947	2.510	3.000	0.779	1.081	3.735	6.968	0.780	2.335
Singletask 1DCNN, No Spectral Trim	1.183	0.465	0.935	2.466	1.809	1.808	1.652	2.366	4.559	0.738	1.798
MLP	1.878	0.596	0.921	3.120	1.765	1.509	1.692	2.232	2.752	0.582	1.705
LSTM	0.927	1.393	1.115	0.809	2.463	0.837	1.319	1.687	2.309	2.775	1.563
PLSR	0.322	0.291	0.725	0.934	3.664	0.977	0.475	1.816	5.049	1.389	1.564
XGBoost	0.477	0.007	1.198	2.251	3.421	1.647	0.333	0.079	2.463	3.654	1.553
Ensemble	0.912	0.328	0.941	1.060	2.630	0.229	0.588	2.085	4.899	0.416	1.409

The results reported are the mean of three runs with different random seeds

**Table 5:** REP for various models on the test set

Model	LMA	N_area_	SPAD	N_mass_	*V*_*cmax*_	*V*_*cmax25*_	*J*	*A*	g_s_	*V*_*cmax25*_/N_area_	Mean
Multitask 1DCNN	7.611	8.446	7.430	8.764	18.723	14.004	10.417	14.485	32.590	15.897	13.837
Multitask 1DCNN, No Spectral Trim	7.026	7.463	6.887	7.610	18.907	14.294	10.252	13.586	31.792	15.243	13.306
Singletask 1DCNN	7.949	6.776	6.666	10.653	18.039	15.184	10.890	15.526	32.761	16.411	14.086
Singletask 1DCNN, No Spectral Trim	7.231	6.120	6.794	8.955	17.452	14.501	10.791	14.983	32.677	15.691	13.519
MLP	7.921	9.550	6.754	10.709	19.925	15.731	12.687	15.954	35.074	15.708	15.001
LSTM	11.337	14.058	9.089	11.222	25.840	17.848	14.338	17.269	37.151	22.766	18.092
PLS	7.076	7.650	7.264	9.179	19.181	17.477	10.639	16.069	34.937	14.350	14.382
XGBoost	9.425	7.849	7.630	10.314	24.261	17.011	13.144	16.320	37.131	19.289	16.237
Ensemble	6.767	6.543	6.655	8.188	16.843	14.549	9.763	13.902	31.965	14.301	12.948

The results reported are the mean of three runs with different random seeds

### Effects of spectral range on model performance

**Fig. 9:**
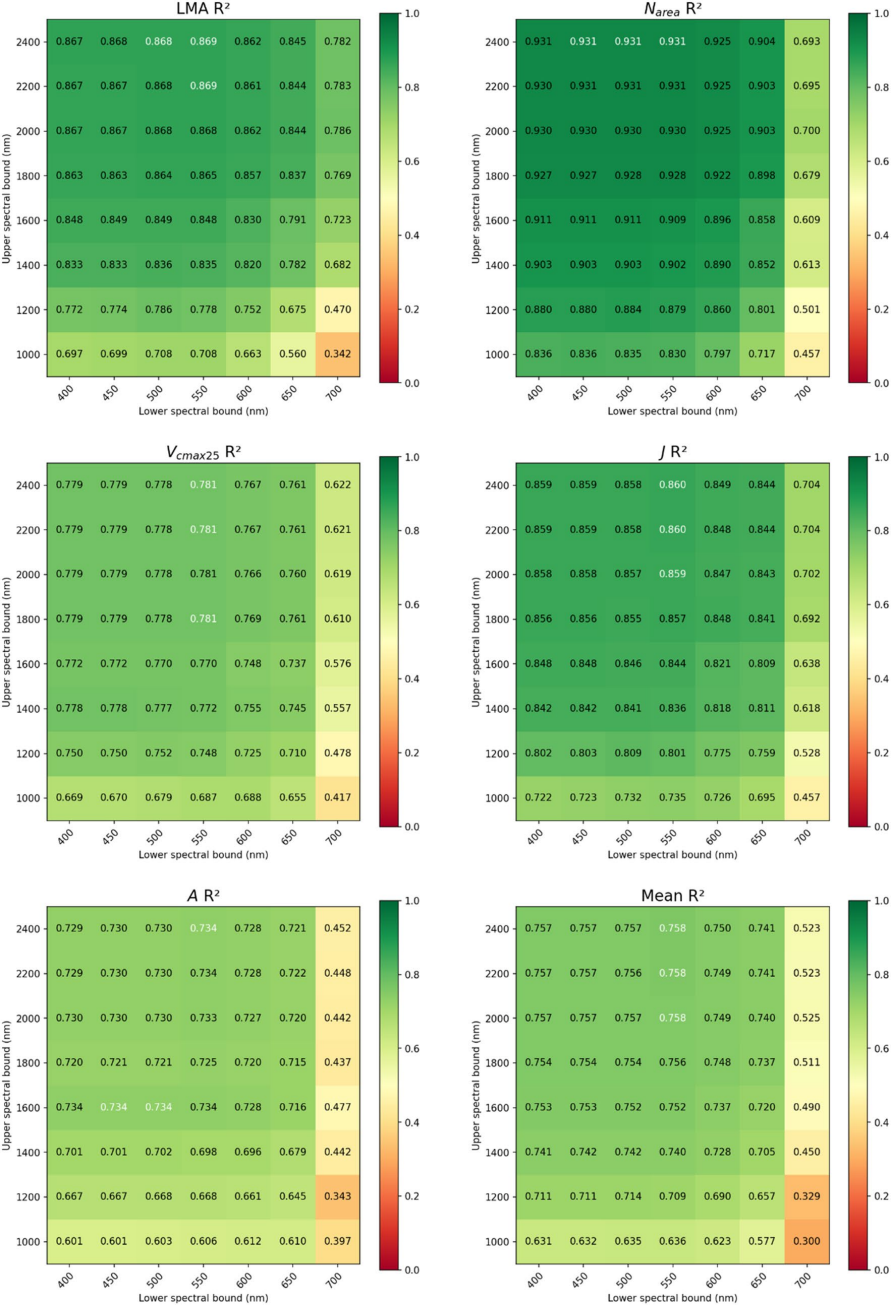
Effect of trimming spectral range of input values for prediction of key traits on the test set using the multi-task 1DCNN model. Values ≥ 95th percentile are displayed in white. Results reported are the mean of three runs with different random seeds

The effects of limiting the spectral range on prediction accuracy of our multi-task 1DCNN model for five predicted traits is shown in Fig. 9. R^2^ values for the correlation between predicted and measured values in the test set varied. Predictions in general were more robust with inclusion of data in the visible/NIR region but wavelengths in the SWIR also improved prediction accuracy. *N*_*area*_ appeared to be the trait least sensitive to omission of SWIR data with R^2^ values ranging from approximately 0.836 to 0.931, respectively when Vis/NIR models (400–1000 nm) are compared with models derived from full range spectra (400–2400 nm). Prediction accuracy for the other 4 traits suffered more from spectral trimming in the SWIR region, although acceptable correlations were still obtained, particularly for *J*, suggesting that utility of these models may be extended to spectrometers with more limited wavelength range.

## Discussion

Machine learning and artificial intelligence have already had a major impact across all aspects of our daily lives, from image recognition in social media to personalized digital media, robotics, and “big data” science. In crop physiology, plant phenomics, and crop breeding, deep learning algorithms such as neural networks have primarily been used in computer vision applications. There has been a large amount of recent work on using deep learning methods to perform image-based phenotyping of plants, in particular using convolutional neural networks (CNN). These methods are useful for learning plant classification, segmentation, detection and other computer vision tasks required in plant phenotyping (Ubbens et al. [37]; Namin et al. [38]; Krause et al. [23]; Zhu et al. [39]).

Despite the popularity of deep learning in plant phenotyping and the proliferation of studies using statistical approaches to derive plant traits from spectral data, these studies have almost exclusively used PLSR [6, 8, 10, 11, 13–16]. One exception is the recent study by Fu et al. [8] that uses an ensemble of six machine learning algorithms to map hyperspectral reflectance measurements to physiological traits for transgenic tobacco plants. The machine learning algorithms they used include fully connected neural networks, support vector machines (SVM), least absolute shrinkage and selection operator (LASSO), random forest, Gaussian process (GP), and PLSR. Using an ensembling technique called stacked regression [40], they showed that the ensemble of the machine learning algorithms outperformed PLSR alone by about R^2^ = 0.1, above a baseline R^2^ of 0.60 to 0.65, based on R^2^ between predicted and observed data in the test set [8]. Our work differs from this study in several ways. The transgenic tobacco material used for the training set in Fu et al. [8] displays a substantially higher range of variation in measured photosynthetic traits than in populations of genetically diverse crop species such as those used here. Generating trait values where the photosynthetic properties have been artificially altered thus provides a potentially easier prediction target for the models. Importantly, natural genetic variation for photosynthetic traits in wheat spans a much smaller range (commonly less than 30% of the mean for a population [4, 12]). Thus, using such an approach to screen for genetic variation in crop photosynthetic performance can be much more demanding of accurate predictive algorithms than detection of transgenic modifications.

Our study presents several novel advances in this state of the art, such as the use of a 1D CNN to extract local spatial patterns from the hyperspectral reflectance data. In addition, this study explores a range of approaches to reduce overfitting of models to the training set, explores multiple traits of agronomic importance extracted from our models, and expands the utility of our models by spectral trimming of training sets and the ability to train a single model for all traits extracted.

As a result of these novel modeling approaches, we found major advantages of deep learning approaches over PLSR:

1. A single deep learning model can be constructed for multiple traits, reducing time and complexity compared to PLSR for model construction and runtime for algorithms.
2. A single neural network can leverage relationships between traits in developing a highly accurate model.
3. Deep learning derived algorithms can cope with data sets of variable spectral range, potentially allowing adaptation of models to different, more affordable spectrometers, even imaging spectrometers [24].

**Fig. 10:**
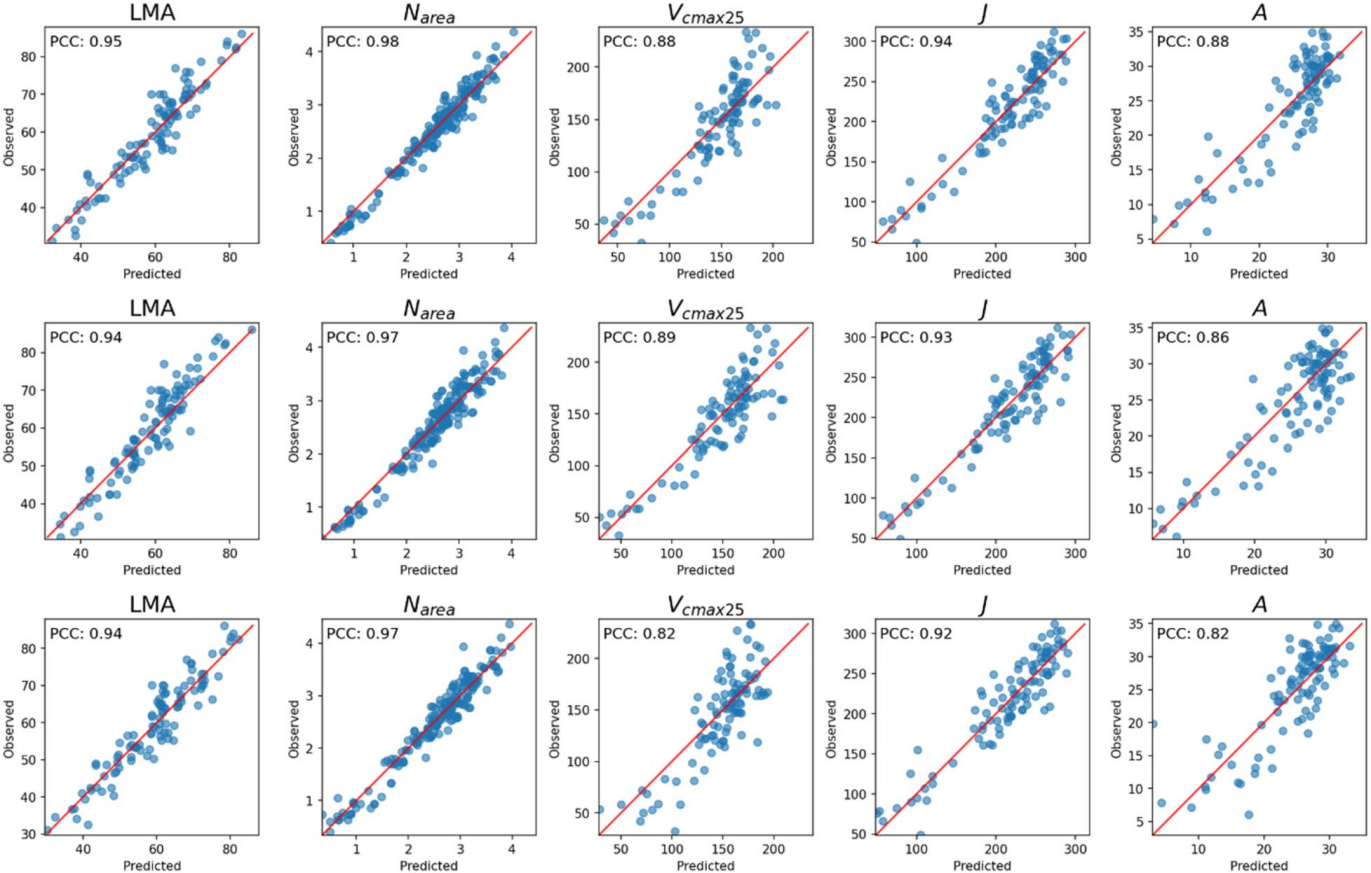
Correlation between key predicted and observed traits on the test set using the ensemble (row 1), multi-task 1DCNN (row 2) and PLS (row 3) models. Pearson Correlation Coefficient, commonly used for ranking purposes, is reported

The accuracy of the 3 best models tested in the current work is shown in Fig. 10 which compares the correlation between key predicted and observed traits on the test set using the CNN ensemble, multi-task 1DCNN and PLSR models. As observed in our previous work [4], the leaf biochemical and structural traits N and LMA were less challenging to predict than the rate of photosynthesis or the modelled parameters *V*_*cmax*_ and *J*. Our multi-tasking deep learning ensemble model produced an overall R^2^ value of 0.79, versus R^2^ = 0.74 produced by PLSR, with considerable improvements in model performance for the photosynthetic parameters *V*_*cmax25*_, *J* and *A*.

A major challenge for spectral prediction of crop traits by machine learning is the difficulty and cost of producing a sufficiently large training set. To generate a training set for the leaf structural and nitrogen traits, leaves must be harvested and dried, measured, weighed and in the case of N, milled and then the material passed through a mass spectrometer [4]. For modelled photosynthetic traits, time consuming gas exchange must be carried out on each leaf in the training set, taking up to 20 min per sample [7]. Indeed, a major attraction of this spectral reflectance method is to reduce the measurement time from hours to seconds for a suite of traits. If training sets are too small relative to the number of spectral bands collected, overfitting of data can limit the capacity for the models to predict into a previously unseen data set. Here we have investigated a number of solutions to this problem, namely optimisation of the number of training epochs and expanding the training set by data augmentation. Both these approaches had significant value and have been incorporated into the model building (Additional file 1).

While a great deal of work has been published on PLSR modelling of spectral data, it is difficult to reproduce these models and use them to predict traits from new spectral data sets as the models themselves are rarely published or made available. To reuse these models, one commonly would have to download the training sets and recreate the models locally, with the corresponding risk that the resulting models are not identical: clearly not practical for most plant biologists. In the current work, we have made the code for the models available to the reader and provided a web application containing stable versions of each model available under a creative commons license. This allows researchers to upload spectral data and predict physiological traits in wheat (Wheat Physiology Predictor (shinyapps.io)) without suffering undue technical challenges, the risk of code or database deprecation, or inaccessible authors. We hope to create a community of users and develop and improve the models and traits predicted as data sets increase in volume and more training sets become available.

### Supplementary Information

**Additional file 1.** Effect of optimsing number of training epochs and expanding training sets by data augmentation on model prediction.

## Data Availability

Training data and models used to derive the results shown in this work are available at https://wheatpredictor.appf.org.au/.
