# Correction: Wheat physiology predictor: predicting physiological traits in wheat from hyperspectral reflectance measurements using deep learning

**DOI:** 10.1186/s13007-024-01153-y

**Published:** 2024-02-24

**Authors:** Robert T. Furbank, Viridiana Silva-Perez, John R. Evans, Anthony G. Condon, Gonzalo M. Estavillo, Wennan He, Saul Newman, Richard Poiré, Ashley Hall, Zhen He

**Affiliations:** 1 ARC Centre of Excellence for Translational Photosynthesis, Research School of Biology, Australian National University, Canberra, ACT 2601 Australia; 2 Agriculture Victoria, 110 Natimuk Road, Horsham, VIC 3400 Australia; 3 CSIRO Agriculture and Food, PO Box 1700, Canberra, ACT 2601 Australia; 4 Australian Plant Phenomics Facility, Australian National University, Canberra, ACT 2601 Australia; 5 Department of Computer Science and Computer Engineering, La Trobe University, Bundoora, VIC 3086 Australia

**Correction to: Plant Methods (2021) 17:108**

10.1186/s13007-021-00806-6

In the original publication of the article [1], the previous link https://plantpredict.shinyapps.io/PredictionShiny/ should have been changed to the new link https://wheatpredictor.appf.org.au/. The original article has been corrected.
