# Supplementary material for: Wheat physiology predictor: predicting physiological traits in wheat from hyperspectral reflectance measurements using deep learning
Source: Plant Methods. 2021 Oct 19;17:108. doi: 10.1186/s13007-021-00806-6 (PMC8527791; doi:10.1186/s13007-021-00806-6)
Supplement: Supplementary file 1 — Additional file 1. Effect of optimsing number of training epochs and expanding training sets by data augmentation on model prediction. [file 13007_2021_806_MOESM1_ESM.docx]

## **Supplemental Data**

**Ablation Study**

An ablation study was performed to measure the impact of model architecture and training design choices, with the R² results presented in Supp Table 1. Dropout was shown to improve the model performance, whereas the addition of spectral trimming yielded a drop in model accuracy. We regard this marginal decrease in performance to be an acceptable trade-off for the flexibility of producing a model capable of accepting input wavelength spectra of varying domains.

| **Model** | LMA | N_area_ | SPAD | N_mass_ | *V_cmax_* | *V*_cmax25_ | *J* | *A* | g_s_ | *V_cmax25_*/N_area_ | Mean |
| --- | --- | --- | --- | --- | --- | --- | --- | --- | --- | --- | --- |
| Full Model | 0.867 | 0.931 | 0.833 | 0.807 | **0.781** | **0.779** | 0.858 | 0.730 | 0.502 | 0.480 | 0.757 |
| No Spectral Trim | **0.887** | **0.946** | **0.857** | **0.855** | 0.776 | 0.770 | **0.863** | **0.762** | **0.526** | **0.521** | **0.776** |
| No Dropout | 0.883 | 0.922 | 0.849 | 0.767 | 0.778 | 0.741 | 0.835 | 0.680 | 0.387 | 0.364 | 0.721 |

*Supplementary Table 1 – Ablation study results for the multi-task CNN; R² values reported. Results reported are the mean of three runs with different random seeds.*
